# Cognitive architecture and behavioral model based on social evidence and resource constraints

**DOI:** 10.1186/s40708-026-00294-1

**Published:** 2026-03-05

**Authors:** Anton Kolonin

**Affiliations:** 1 The Artificial Intelligence Research Center, Novosibirsk State University, Pirogova 1, 630090 Novosibirsk, Russia; 2 SingularityNET Foundation, Baarerstrasse 141, 6300 Zug, Switzerland

**Keywords:** Behavioral model, Belief system, Cognitive architecture, Resource constraints, Social evidence

## Abstract

The cognitive architecture presented in this paper is expected to be able to explain certain aspects of human behavior, guide the development of artificial intelligence agents, and align the behavioral patterns of the latter with the former. The architecture is based on the principle of social proof or social evidence, including the principle of resource constraints. It includes the concept of a hybrid knowledge graph that encompasses both symbolic and sub-symbolic knowledge. This knowledge is divided into functional segments for fundamental, social, evidential, and imaginary knowledge, and is processed by an inference engine and a memory storage system that are aware of and manage resource constraints. The architecture and behavioral model derived on its basis are expected to be used to design artificial intelligence agents and decision support systems that are consistent with human values and experiences based on the alignment of their belief systems, capable of implementing decision support systems for practical applications. It can also be proposed for modeling human behavior individually or in a group, for psychological treatment, online security, and community management.

## Introduction

### Significance

We propose the development of a cognitive architecture and an algorithmic model of belief formation and decision-making based on the principles of social evidence or social proof and resource constraints. To our knowledge, this has not been done together before and is the novelty of our work. We expect that this will be of value to researchers designing or building artificial intelligence (AI) systems and agents that match human values and experiences based on the alignment of their belief systems, capable of implementing decision support systems for practical applications. Such AI applications can be considered trustworthy because they are based on interpretable cognitive and behavioral models. Moreover, since the consideration of cost and time factors is built into the proposed inference engine and memory storage design, it can serve to build scalable and time-critical AI solutions. Furthermore, we expect that the cognitive and behavioral model based on the proposed architecture can be used to model human behavior individually or in groups, create tools and frameworks for psychological treatment, online security applications, and community management solutions at various scales.

### Motivation

The motivation of our study lies in Nick Bostrom’s work in the late 1990s and early 2000s, addressing the aspects of creating AI systems that align with human values [5]. An extension of this work and practical considerations can be found in more recent studies, where not only empathy and alignment with human values are considered as the basis for an AI agent architecture, but also the principle of resource constraints in terms of energy consumed and time allocated to the decision-making process is taken into account. This more recent work proposes the concept of a resource-constrained cognitive model based on social evidence for empathy-driven AI [22].

Further developments point to the enormous importance of AI systems in meeting modern needs for empathy, compassion and trust, particularly in areas such as healthcare [20].

Moreover, from the perspective of a general theory of general intelligence, as Goertzel [11] suggests, the pragmatic "patternist" view argues that broad compassion and empathy are necessary qualities of all intelligent beings, and therefore should be implemented in artificial intelligence systems as well.

From this point on, we are looking for a stable foundation that would justify the necessary cognitive architecture that supports the corresponding behavioral model of an artificial intelligence agent.

### Background

A mathematical model of individual behavior was developed in Vladimir Lefebvre’s book, The Algebra of Conscience [30]. Although the book is not supported by actual field data, it presents a computable behavioral model that can predict the behavior of a mathematical model of an individual or a small group of individuals, given basic a priori data such as the core elements of the individual’s belief system.

The social nature of human decision making, individually and in groups, was described in detail by Cialdini in [6] based on numerous field studies, where the author provides extensive evidence that conclusions for forming a system of beliefs or decisions to take certain actions are based on what he called "social proof"—reliance on the beliefs and decisions made by people around.

**Fig. 1:**
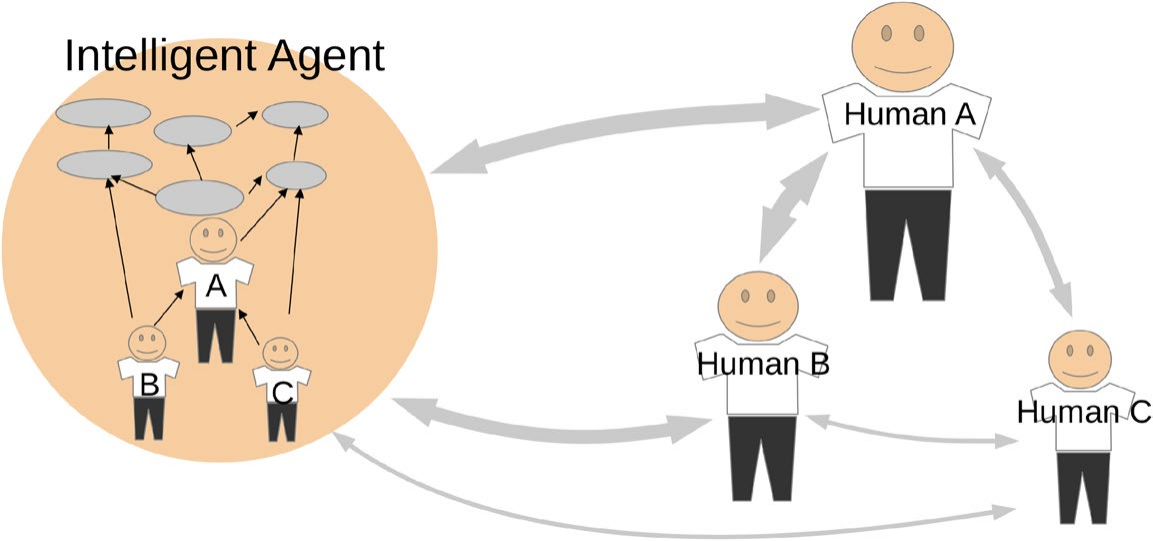
The belief system of an AI agent (left) includes a model of the external world, including the social beings around it (right), as well as subjective projections of the social relations between them, as well as their own belief systems

Attempts to link the quantitative computational mathematical approach and qualitative field data were made later in Kolonin [21] and Kolonin et al. [29], where Robert Cialdini’s notion of "social proof" was transformed into the concept of "social evidence" involved in the process of probabilistic logic, according to Goertzel et al. [13] and Vityaev et al. [38] within the framework of Petr Anokhin’s "Theory of Functional Systems" [32]. In these attempts, an agent’s belief system is described in terms of a computable weighted graph that probabilistically describes a model of the external world for the agent, where the model includes not only the adjacent physical environment and the social beings in the neighborhood, but also the subjective projections of the belief systems of these neighboring agents onto the former agent’s own belief system, as shown in Fig. 1.

The representation of the belief system as well as procedural knowledge for the past and possible actions, including experiences recorded in episodic memory can be stored in generalized hyper-graph structures, where not only the "link" can be associating more than two or three linkable entities, but the link can serve as an entity itself, being linked with the other entities or links, potentially being part of multiple directed and recurrent graphs or heterarchical networks, according to Goertzel et al. [12].

**Fig. 2:**
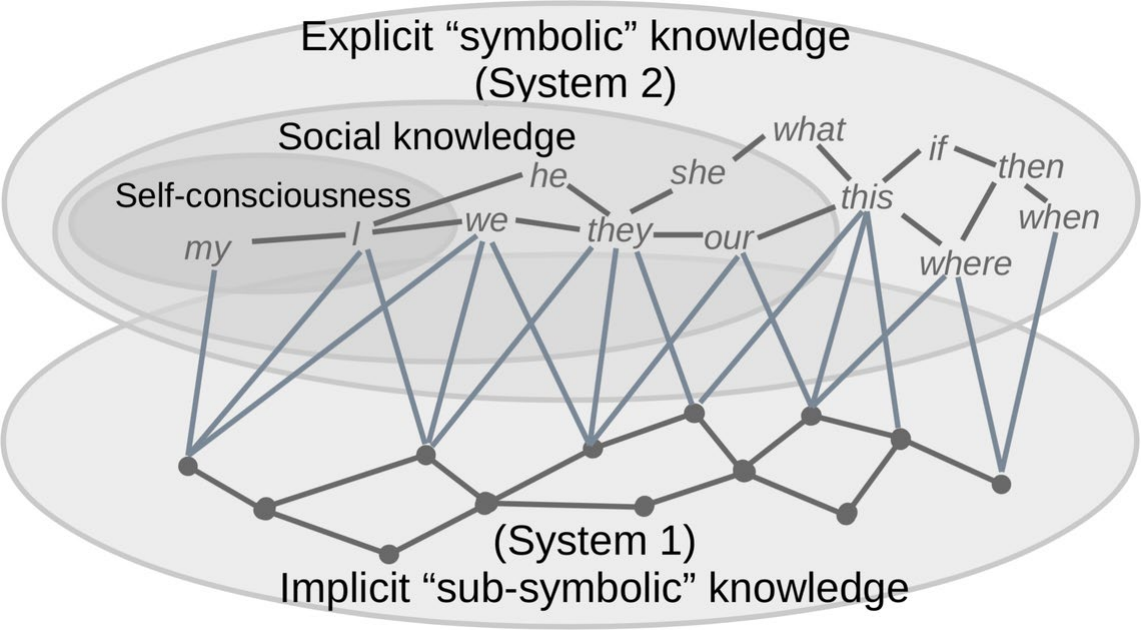
Multi-layer symbolic/sub-symbolic heterarchical hypergraph linking explicit "symbolic" knowledge for logical reasoning (Kahneman’s "System 2," top) and implicit "sub-symbolic" knowledge for intuitive belief and action (Kahneman’s "System 1," bottom)

Such a generalized hypergraph, consisting of heterarchical networks spanning several layers of parallel heterarchical networks, might span subgraphs corresponding to the explicit or symbolic parts of the agent’s knowledge, as well as subgraphs corresponding to its implicit or sub-symbolic knowledge. This might be well-mapped to Daniel Kahneman’s concept of two systems of thinking, according to Kahneman [19], where the former subgraph would correspond to Kahneman’s "System 2" of slow explicit and logical reasoning, and the latter one would correspond to "System 1" of fast intuitive inferences and decisions. An example of such a hybrid interconnected hypergraph is shown in Fig. 2. Moreover, the research conducted by Cisek [7] shows that the actions of these two systems of thought, realized in the dynamics of the two corresponding networks, are in many ways complementary, since they operate in the same context to achieve the same goal on a competitive basis.

While the probabilistic logic implementations presented in Goertzel et al. [13] and Vityaev et al. [38] can perform inference based on uncertain and incomplete knowledge, non-axiomatic logic and the corresponding reasoning framework, originally proposed by Wang [39] as the essence of intelligence, include a full accounting of both insufficient knowledge and resources. That is, according to Wang [41], inference engine must recognize and take into account not only the unreliability, uncertainty, and insufficiency of the data available for inference, but also the limited time and energy resources allocated. This means that the inference engine must be able to find optimal strategies to minimize the energy consumption of the inference process according to the priorities of the inference tasks and provide the best possible results within the given time frame.

Another perspective on resource accounting is provided by Sleator and Temperley [33], where the language model replaces probability with "cost", so that, according to this model, predictions of optimal parsing trees during natural language processing are selected based on cost minimization. For this purpose, the underlying language model is expected to support the cost associated with each grammatical link, or conjunction or disjunction of links that make up the model.

Recent work proposes to take into account the cost and minimization of the energy expended in inference, contrasting probability-based and energy-based methods: "instead of predicting a single most probable event, we can let the model represent the dependency between variables through the energy function", according to Dawid and LeCun [9], consistent with the "Free Energy Principle" posed by Friston [10]. The latter and related work [31] propose the "Free Energy Principle" as the basis for a unified theory of mind, brain, and behavior that links maximization of the probability of a correct prediction with minimization of so-called "free energy."

**Fig. 3:**
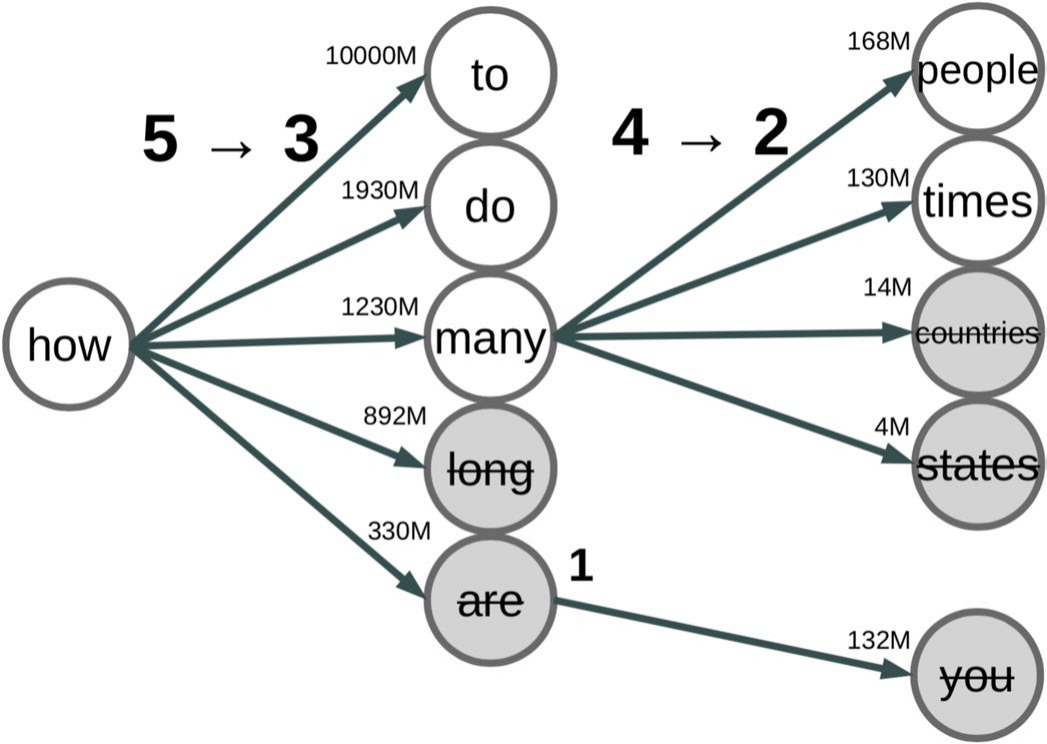
The "Transition Freedom" (*TF*, bold numbers) and transition frequencies represented by a state transition graph. Initially, $TF=5$ for the transition from the state "how" to some next state and $TF=4$ for the transition from the state "how$\rightarrow$many" to another state, such as "how$\rightarrow$many$\rightarrow$people". Storage and decision-making costs can be reduced by lossy graph compression by eliminating low-frequency transitions. After this, $TF=3$ for the transition from the state "how" and $TF=2$ for the transition from the state "how$\rightarrow$many"

A practical study of different scoring functions based on condition probabilities and so-called "freedom of transition" for text segmentation models, according to Wrenn et al. [44], points to the superiority of models based on "freedom of transition" in improving the accuracy of text segmentation. On the other hand, minimizing the overall "freedom of transition" within the whole model, as shown in the Fig. 3, can be interpreted as a way to compress the model itself, minimizing the cost of its storage and the cost of inference using it at the same time. The figure is based on a Google search for the corresponding N-grams, showing the number of possible results returned (small numbers in the millions). The transition from the unigram "how" to bigrams like "how$\rightarrow$to", "how$\rightarrow$do", and "how$\rightarrow$many" has an initial $TF=5$, the transition from the bigram "how$\rightarrow$many" to trigrams like "how$\rightarrow$many$\rightarrow$people" and "how$\rightarrow$many$\rightarrow$times" has an initial $TF=4$. Model compression (pruning) based on the conditional probability threshold on each transition link reduces the first *TF* from 5 to 3, and the second *TF* from 4 to 2.

A subsequent multicultural study that examined human languages as efficient codes of communication found that a metric of "freedom of transition", called "transition freedom" (TF) in Kolonin [26], can not only provide accurate predictive models but also govern the structure of human language. That is, human languages such as Chinese, English and Russian were found to be optimally structured in terms of minimizing entropy and maximizing information compression at the same time.

While a predictive model of the world surrounding an agent, be it a human, an animal, or an artificially intelligent being, can be optimized based on maximizing probability or minimizing computational costs, or both, the agent’s behavioral activity can be described based on Anokhin’s theory of functional systems, according to Red’ko et al. [32], and implemented in an appropriate cognitive architecture, such as that proposed by Vityaev and Demin [35].

The development of such an architecture, according to Vityaev et al. [36], is based on the assumption that maximizing the accuracy of predictions in order to build a model that is beneficial to the agent is evaluated in terms of a hierarchy of goals and objectives imposed on the agent by evolution, the environment, and its own "state of mind". That is, the usefulness and value of a prediction and its chance of being reinforced in the experiential learning process are based on the agent’s belief system and individual values at the time of the prediction. Notably, since minimizing energy consumption and the periodic vital need to make timely decisions are parts of the agent’s value hierarchy, these factors have to be naturally included in the hierarchy of goals and objectives.

Another aspect of cognitive architecture that should be considered is the need for the ability to adapt to changes in its model based on new situations and environmental changes, such as the migration of new knowledge with sufficient subjective importance from the agent’s short-term memory (STM) to its long-term memory (LTM). While architectures based on large language models (LLMs) currently lack this capability, there is research into how these architectures can be extended to allow models to self-evolve over time [18]. The different functional roles of these two types of memory are thought to be important for human cognitive operations [3], and this is an area of research exploring how the different types of memory in the human brain can be used in modern cognitive architectures for artificial intelligence [45].

When speaking about the agent environment, for modern humans and practical AI systems it is necessary to take into account its essentially social nature. That is, most of the agent’s encounters with reality, the formation and testing of its cognitive process and behavioral model involve other agents with their actions and messages made on the basis of their own belief systems and behavioral models. The importance and influence of such social dynamics, mathematically grounded by Lefebvre [30] and phenomenologically investigated by Cialdini [6], have recently been presented in the latest study of belief networks, attempting to build an integrative theory of belief dynamics at the individual and social levels [8].

## Cognitive-behavioral architecture and model

Based on the motivation and premises outlined above, we propose a cognitive architecture for implementing a behavioral model based on the principle of social evidence or social proof including the principle of resource constraint. The architecture comprises a hybrid knowledge graph, structured as described below, including both symbolic and sub-symbolic knowledge and supported by short-term memory (STM) and long-term memory (LTM) subsystems. The latter sub-systems are equipped with processes for moving knowledge graph elements between the sub-systems and removing them during the forgetting process, as well as an inference engine for computing the probabilities of elements in STM given the current operational context. The behavioral model involves mapping perceptual inputs taken from the external environment and recorded as evidence inputs to actions directed against the environment, based on the inference engine’s evaluations of these actions in STM, according to the graph structure and computational scheme described further.

Given that the architecture can manage the cognitive process of formation and dynamic changes of the agent’s belief system, as well as its exploratory and proactive behavior, we can call the entire architecture cognitive-behavioral. In turn, the model underlying the architecture at the conceptual level and any specific model developed by the agent during its cognitive development and proactive interaction with the environment can be called a cognitive-behavioral model. The difference between them is that the architecture defines principles of practical implementation, such as specialized but overlapping segments of a hypergraph encompassing implicit and explicit knowledge together with STM and LTM, while the model represents principles of inference involving subgraphs of concepts of belief system, world knowledge, and procedural knowledge located in these graphs.

In the following design, we use terms like "knowledge", "graph", and "concept" loosely, in terms of a generalized "hypergraph" encompassing symbolic and sub-symbolic knowledge described in Goertzel et al. [12], as shown in Fig. 2. Accordingly, a "concept" may not necessarily refer to a low-level terminal "atomic" concept, such as a token or a letter. Instead, any "atomic" concept in the "hypergraph" may be considered a subgraph. For example, a token can be rethought as a subgraph of morphemes and letters, while a letter can be interpreted as a subgraph of strokes, and so on. Moreover, concepts in a belief system can be either simple things like the ideas of light and dark or complex concepts like the idea of quantum physics, while concepts in procedural knowledge can be either simple triggers acting on certain muscles or servomotors or rather complex patterns like the rules of a baseball game.

### Overall architecture

**Fig. 4:**
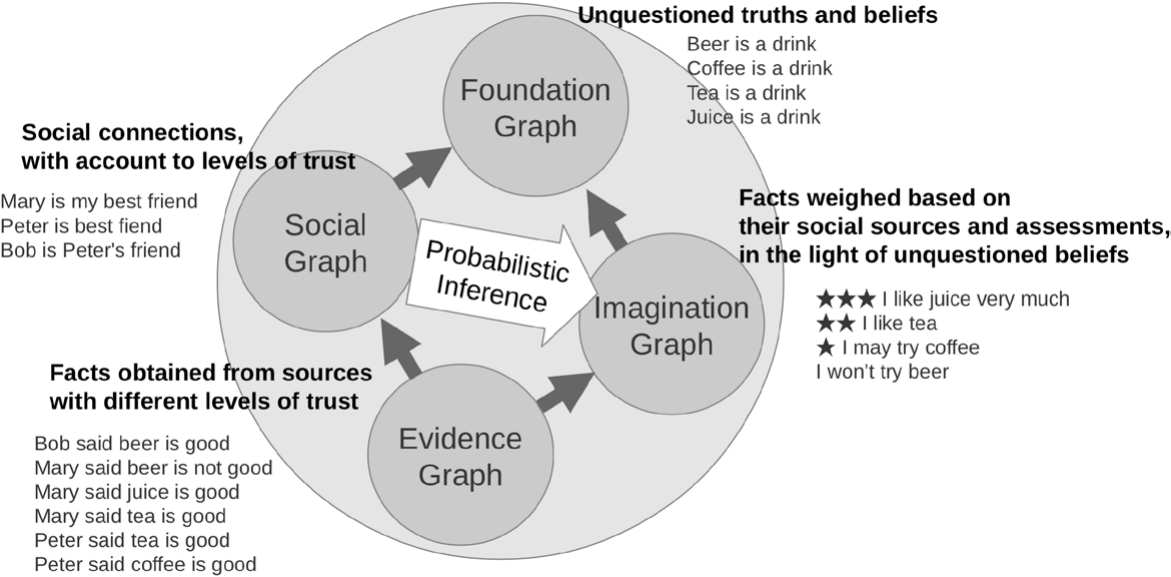
Segmentation of the knowledge graph of a cognitive-behavioral agent model into four subgraphs: the Foundations Graph, which contains core beliefs, the Social Graph, which provides social references for evaluating facts received through the Evidence Graph, and the Imagination Graph, which contains a dynamic worldview derived from the other three graphs

The key principle of the architecture and model is the classification of knowledge, including the belief system, world knowledge, and procedural knowledge, from the point of view of the agent and its inference engine, into four functionally distinct subgraphs, as shown in Fig. 4. In fact, the boundaries between subgraphs may be blurred, and in different contexts the same piece of knowledge may be considered to belong to one or another subgraph, but this distinction is nevertheless important from the point of view of understanding and implementation.

The top subgraph in Fig. 4, called the "Foundation Graph", contains unquestioned truths and beliefs, including core values and automated procedural knowledge at the top of the knowledge hierarchy—no assessment of reliability and confidence is required for this, and so the computational cost of maintaining it is low.

The leftmost subgraph in Fig. 4 contains social connections to social trust accounts—social connections, sources, and referees providing facts, feedback, and evaluation of facts and actions, including the agent’s own actions. It represents the agent’s internal "Social Graph", which can be used as a reference for evaluating facts to believe or not, and actions to take or avoid, according to Cialdini [6].

The bottom subgraph in Fig. 4, called the "Evidence Graph", contains facts from everyday interactions with the physical and social environments, as well as the actions taken by the agent with respect to these environments. The facts can be obtained from sources with varying levels of credibility, including observations coming from the outside world, predictions made by sources in the social environment, and personal exploratory, proactive, or prospective actions performed or expected by the agent possessing the model.

The right subgraph in Fig. 4, called the "Imagination Graph", contains uncertain or probabilistic knowledge that is not part of the agent’s belief, but is dynamically inferred on the basis of the latter belief in the context of social sources (from the "Social Graph") providing facts and predictions (from the "evidence graph") and social referees evaluating the agent’s own actions taken or expected. The facts and actions here are weighted in the context of social sources and referees providing and evaluating them, constructing the agent’s current representation of the dynamic world, its place in it, and the actions it is expected to take toward the world.

The "Probabilistic Inference" mechanism in the middle of the cognitive architecture in Fig. 4 is connecting parts of the entire knowledge representation model, such as perceptions of the external world and planned actions with respect to it. The results of the latter inference are stored in a subgraph of the "Imagination Graph" inferred from the other subgraphs according to the formulas presented later in this paper, using one of the inference systems such as those described in [13, 40, 41], or [38]. That is, the engine dynamically constructs the "Imagination Graph" from the "Foundation Graph" using the "Evidence Graph" as input in the context of the "Social Graph".

**Fig. 5:**
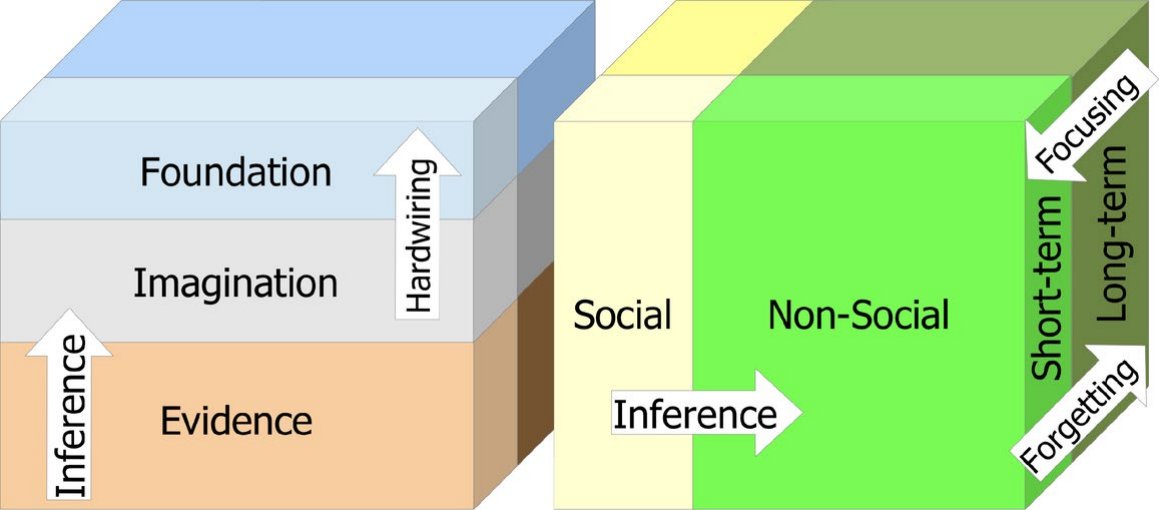
Three different approaches to segmenting the knowledge representation model into overlapping layers. Bottom-to-up: from the raw "Evidence" data at the bottom, through the dynamic worldview as an "Imagination" inferred from that data, to the "Foundation" beliefs. Left-to-right: from "Social" knowledge providing social references to the "Non-Social" factual and inferred knowledge derived from those references. Front-to-back: "Short-term" memory for operational activity, retrieving knowledge elements from "Long-term" storage, where the novel knowledge is memorized and forgotten later if not needed

Since not all elements of everyday evidence, nor most actions taken or planned by an agent, can be explicitly evaluated by social referees, the expected subjective evaluations of them in terms of whether the agent’s current belief in its social peers can be consumed, so there may be recurrent involvement of each element of the agent’s knowledge in different graphs in different roles. This is shown in the Fig. 5, where the same “knowledge cube” can be cut from three different points of view.

First, the knowledge may be part of "Evidence", "Imagination" or "Foundation", having the volume of the belief and procedural knowledge sliced based on its reliability—with unconditionally and statically believed "Foundation" at the top, contextual and dynamically perceived "Imagination" in the middle, unreliable and occasional stochastic stream of facts and actions as "Evidence" at the bottom.

Second, the same volume of knowledge can be divided on the basis of having either "Social" or "Non-Social" nature (left and right in the Fig. 5), being either part of "Social" knowledge, representing social sources and referees, relations with them and subjective projections of relations between them and their own internal beliefs or the rest of "Non-social" knowledge about physical world.

Third, the knowledge can be divided into layers based on its presence in “Short-term” or “Long-term” memory (front and back in the Fig. 5) as it can be either in “Short-term” memory in the focus of attention and operational context, or remain in “Long-term” memory and outside the focus of attention.

These different ways of partitioning the whole graph, represented as layers in Fig. 5, may involve the same pieces of implicit or explicit knowledge, so that some of it may be "Social", residing in the "Foundation" layer of "Long-Term" memory, while others may reside in the "Non-Social", "Imagination", and "Short-Term" layers simultaneously. Moreover, the links in the "Social" subgraph used to infer any elements in the "Imagination" subgraph according to the formulas in the next section may themselves be the subject of inference, so that situations like "Yesterday I assumed that she was quite sure that he liked me to steer the boat" involve recursive inferences involving multiple subgraphs recurrently.

**Fig. 6:**
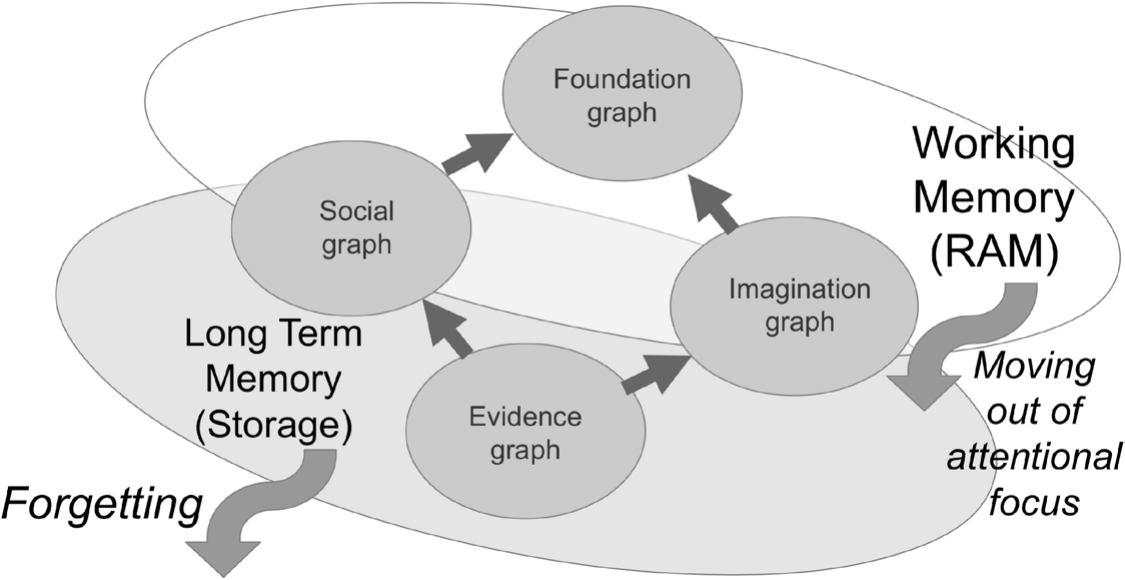
Long-term memory (LTM) is used for permanent storage of information (e.g., on disk), while short-term memory (STM) is used for the agent’s working memory (e.g., RAM). Resource constraints apply differently to these memory types: knowledge items are removed from the STM’s view if they don’t fit its capacity, and they are completely removed from LTM through the forgetting process if they are no longer needed or if storage capacity is exhausted

The implementation of the "Short-term" ("Working") and "Long-term" ("Storage") memory layers can be thought of in terms of a fast, low-capacity graph store for the former (STM) and a slow, high-capacity store for the latter (LTM). For example, random-access memory (RAM) can be thought of for the STM and disk storage for the LTM. For a more practical example, one could use an in-memory graph or vector database for the STM and a relational database for the LTM. All knowledge that the agent currently possesses is stored in the LTM, and only the part of it specific to the current operational context, including the core part of the "Foundation" subgraph, recently perceived evidence in the "Evidence" subgraph with executed responses and actions, and hypotheses, expectations, and planned responses and actions in the "Imagination" subgraph, is stored in the STM. In its turn, LTM stores all historically recorded evidence and action logs as "episodic memory", the full social graph, and the full worldview learned to date in the belief system, including "trusted knowledge" in the "Foundation" subgraph and "uncertain knowledge" in the "Imagination" subgraph, see Fig. 6. "Working" memory (STM) corresponds to an expensive, high-throughput store for dealing with a limited amount of temporary knowledge and actions relevant to the current "operational context." Long-term memory (LTM) corresponds to an inexpensive, low-throughput memory that stores the entire belief system, world knowledge and procedural knowledge possessed by the agent. Resource constraints imposed by the available capacities of both memories are associated with the displacement of situationally irrelevant fragments of knowledge from STM to LTM and the complete forgetting of irrelevant or outdated knowledge being removed from LTM.

Maintaining the “two memory stores” construct described above requires three processes: (a) a “Focusing” process that transfers certain LTM contents into "attentional focus" in STM based on the current operational context present in the latter; (b) an “Unfocusing” process that moves contents out of the STM "attentional focus" to make room for more relevant contents; and (c) a “Forgetting” process that involves “compression” of knowledge in LTM by eliminating pieces of “Evidence” and segments of the “Foundation,” “Social,” and “Imagination” subgraphs that are unlikely to be used in the future or have not been used for a long time, or under both criteria. Importantly, “Unfocusing” in respect to newly acquired knowledge moving it out of the operational context may also involve “remembering” it in LTM as it is acquired, since new portions of any subgraph newly created in STM during the inference process may not be present in LTM at the time of the unfocusing and therefore must be preserved by continually storing the valuable new knowledge in LTM.

The limitations imposed by limited memory and computing resources used to store and output data can be addressed at the architectural level using the processes defined above, as illustrated in Fig. 6. The impact of limited resources for STM may result in less evidence and context information being placed in the STM for inference, so the inferred decisions and actions may be imperfect compared to those inferred on a more expensive system with more memory used for STM. Alternatively, for this case, implementing caching logic for inference may slow down the inference process by iteratively focusing and unfocusing on different segments of the subgraph being inferred, so that even a very accurate solution may be inferred at the expense of a long time allocated to compute it. Moreover, one of the two proposed inference strategies, such as "fast but incomplete" or "slow but complete" ones, can be selected based on another constraint imposed as "time to decision", so that the first strategy is selected under time pressure, while the second one can be used under relaxed conditions. It should be noted that the decision about which inference to use in which situation can be a matter of the inference itself, performed on the fly by the inference system in self-managed mode. This can be done using an inference control system that will manage the mode and priority of the inference process under resource constraints according to Wang [41].

The constraints imposed by the capacity of persistent memory to store LTM data are overcome by the process of "Forgetting". The consequence is that a system with insufficient LTM capacity will discard less probable and less frequently used knowledge that was acquired a long time ago in a particular environment where this knowledge was relevant, assuming that such an environment may never be encountered again. However, this can become a problem if the environment changes back and forgotten knowledge can become relevant if preserved. That is, a system with greater LTM capacity will be more general in dealing with all possible environments that it has learned to deal with in the course of its evolution. In contrast, a system with less LTM capacity will be more specialized in dealing with a smaller number of environments that match its LTM capacity, and exposure to new conditions in the world will require a new adaptation from scratch.

The latter architectural features, related to the constraints of available memory, allow us to create a universal solution based on an AI agent that can dynamically upscale or downscale the system on the fly without losing its core intelligence, but simply making it either more accurate, general purpose, and expensive when adding resources, or less accurate and more specialized but cheaper when minimizing use of resources.

The above discussion uses terms like "knowledge" and "graph" without distinguishing between their explicit (symbolic, Kahneman’s "System 2") and implicit (sub-symbolic, Kahneman’s "System 1") components, assuming that both are just different kinds of knowledge, all of which can be stored in some hypergraph database as shown in Fig. 2. Moreover, the reference to "focus of attention" above refers to both kinds of knowledge in a purely technical sense, with STM drawing to its "attention" and loading into itself only the vital segments of LTM that are relevant to the current operational context within the environment and that fit the capabilities of STM. In other words, the loose use of the "focus of attention" term in our discussion can be seen as synonymous with the "operational context". This still allows for multiple specific points or spots of "attention" in STM to be dealt with by the inference engine.

### Computational model

The proposed computational model, based on the discussion above, can be briefly described in the following terms and definitions at the conceptual level, while a specific implementation may require adjustment of a specific inference model, such as one of the probabilistic or non-axiomatic logic systems mentioned above: [13, 38, 39]. The following formulas are defined in terms of the indices of the elements of the knowledge hypergraph described in the previous section, together with the values and properties of these elements. An agent in these definitions can correspond either to a human whose behavior we are trying to describe or to an artificially intelligent system whose behavior we are trying to design. We suggest that the same inference logic can be used to evaluate the strength or expression of reliability regarding concepts as pieces of "evidence" to be believed or ignored, as well as the strength and expression of desire and will to carry out particular "intentions".

Given that the agent’s world model is supposed to maximize the predictability of the world according to [9, 10, 31], and the agent’s behavior is supposed to maximize confirmatory feedback from the environment according to Red’ko et al. [32], we assume that the assessment of the reliability of presented evidence or the adoption of planned actions by social referees from the agent’s point of view can be subject to prediction and inference, like everything else.

*i*—index of the subject of consideration from whose point of view an element of the belief system should be assessed in terms of reliability or an action should be assessed in terms of the necessity of its performance.

*j*—index of the concept to be assessed for reliability and relevance, such as a piece of evidence exposed to an agent, or an action to be considered as preferred for execution ("exposed evidence").

*l*—index of the agent’s belief element in the "Foundation" graph of size *L* ("personal preference base").

*k*—index of the agent’s correspondent in the "Social" graph of size *K* ("social reference base").

$B_{il}$—quantitative evaluation of the mental attachment of agent *i* to its belief system item *l* or the degree of its inclusion in the agent’s "Foundation" graph ("personal preference"). In a simplified form, it can be quantified as 0 or 1, where 0 means that element *l* is not part of the "Foundation" graph belonging to agent *i*, and 1 means that it is part of it. In a more complex implementation, it can be a number in the range 0 to 1 indicating the strength of trust given by *i* to *l*. Its value can be determined using the dynamics of recursive cost optimization and probabilistic inference discussed further.

$S_{ik}$—social attachment of agent *i* to another agent *k* ("social reference"). In a simplified form, it can be quantified as 0 or 1, where 0 means that agent *k* is not part of agent *i*’s "social" graph, and 1 means that there are social relations between agents. In a more complex implementation, it can be a number in the range 0 to 1, indicating the degree of social trust of *i* in *k*. As with the previous parameter, it is dynamically determined by the inference and optimization of the system’s knowledge structure under the load of an increasing flow of incoming evidence through the "Evidence" graph.

$E^B_{ijl}$—agreement or compatibility of concept (action) *j* with belief item *l* in the mind of agent *i* ("believed evidence" or "believed intention"). It can either be stored in memory ("cached") based on previous experience, or computed recursively by the inference engine. For example, if *j* is an action and *l* is a goal, this will be the probability of achieving goal *l* given *j*. If *j* is a piece of evidence (e.g., a forecast of rain given a sunny sky) and *l* is part of a core belief possessed by agent *i* (e.g., "it never rains when it’s sunny"), this will be the subjective probability of *j* given the constraints of *l* from *i* perspective.

$E^S_{ijk}$—approval or confirmation of concept (action) *j* by agent *k* in view of *i* ("social evidence" or “socially justified intention”). As with the previous parameter, it may be cached in memory permanently or evaluated dynamically, depending on the implementation and resource optimization policy. For example, if the exposed concept *j* is "known" to have been communicated by *k* or known to have been accepted by *k* previously, it will be 1. Otherwise, for another example, if it is a planned action that has never been exposed to *k*, the probability of *j* being accepted by *k* may be evaluated by the inference engine.

$E_{ij}$—quantitative evaluation of the subjective reliability of a concept or preference to perform an action identified by *j* on behalf of an agent specified by *i*, as a product of "believed evidence" and "social evidence" ("believed social evidence"—what we think, or "believed socially justified intention"—what we plan to do).

$$\begin{aligned} E_{ij} = \sum _{l=1,L}(E^B_{ijl} * B_{il}) * \sum _{k=1,K}(E^S_{ijk} * S_{ik}) \end{aligned}$$

The above formula is presented in conceptual form. To bring $E_{ij}$ and $\widetilde{E}_{ij}$ into a reasonable range [0.0, 1.0], appropriate normalization is required using mental attachment $B_{il}$ and social attachment $S_{ik}$ as weights to calculate the reliability or preference of a concept or action as a weighted sum of its believed $E^B_{ijl}$ and social $E^S_{ijk}$ evidences of concepts or justifications of intentions, as follows.

$$\begin{aligned} \widetilde{E}_{ij} = \frac{ \sum _{l=1,L}E^B_{ijl} * B_{il} }{ \sum _{l=1,L}B_{il} } * \frac{ \sum _{k=1,K}E^S_{ijk} * S_{ik} }{ \sum _{k=1,K} S_{ik} } \end{aligned}$$

The above normalization scheme, applied to common and socially grounded concepts and intentions, is consistent with how the reliability of factual knowledge is assessed using the "revision rule" of Goertzel et al. [13] and Wang [40], where the formula for the "strength" of Goertzel et al. [13] or the "frequency" of Wang [40] *F* of a generalization of a proposition is derived from the "strengths/frequencies" $f_j$ and the "confidence" $c_j$ of several instances of the proposition based on *J* observations. In the formula below, the reliability of a generalized fact $F_{rev}$, called its "strength" by Goertzel et al. [13] or its "frequency" by Wang [40], can be estimated as a similar weighted sum of the "strengths" or "frequencies" of the supporting observations $f_j$, weighted by their "factual evidence" $D_j$, derived from their "confidence" $c_j$, as $D_j = c_j$ by Goertzel et al. [13] or as $D_j = c_j / (1 - c_j)$ by Wang [40].

$$\begin{aligned} F_{rev} = \frac{ \sum _{j=1,J}f_j * D_j }{ \sum _{j=1,J}D_j } \end{aligned}$$

Accordingly, the same approach can be applied to the probabilistic measure of the reliability of an observable or factual knowledge on a set of observations as presented in Vityaev and Pak [37], where the probabilistic measure $\nu$ of the reliability of a fact is based on the ratio of elements *g* in a set *G* of observations that satisfy a set of conditions $\Phi$ representing a rule confirming the fact. That is, if the evaluation of all conditional formulas $\Phi$ for each observation *g* has the same level of evidence, this can be written as $\nu = \sum {\Phi _g} / G$. However, if the "factual evidence" of measurements *g* in *G* differs, this can be written as a weighted average, similar to the "revision rule" mentioned above, as follows.

$$\begin{aligned} \nu = \frac{ \sum _{j=1,J}\Phi _g * D_j }{ \sum _{j=1,J}D_j } \end{aligned}$$

Based on the above, we conclude that the personal or factual value of a concept or intention can be estimated in the measurable range [0.0, 1.0] as a weighted sum of various social assessments, personal beliefs, or factual confirmations, weighted by the corresponding evidence values. We therefore propose that socially and personally grounded subjective knowledge and behavior, evaluated according to $\widetilde{E}_{ij}$, can be combined with the factual knowledge base based on statistical measures of evidence, according to one of the chosen paradigms, such as non-axiomatic [39, 40] or probabilistic [13, 38] logic.

Moreover, the $\widetilde{E}_{ij}$ formula above may represent an elementary computational kernel that can be applied to each piece of knowledge in the "operational context" of STM recursively, in a loop until the end of the recurrent dependence is reached, or the change in quantitative estimates stabilizes in a steady state, or the inference process is terminated due to a deadline in the "limited time" allocated for the decision-making process. In this recursion, the estimates $E^B_{ijl}$ and $E^S_{ijk}$ can be evaluated in the same way as $\widetilde{E}_{ij}$ in their own contexts iteratively.

Given the constraints of "limited memory" and "limited computational power", whatever that means for humans and artificial agents, according to Wang [39, 41, 42], a change in a particular part of the belief system, world view or procedural knowledge to be evaluated for $\widetilde{E}_{ij}$ should be processed based on its computational priority by a scheduling or prioritizing mechanism built into the inference engine, according to further definition of inference cost $\widetilde{C}_{ij}$, where the "cost", originally defined by Sleator and Temperley [33] can be assessed corresponding to the definition of $\widetilde{E}_{ij}$ and respective graph traversal logic during the inference.

The concept of "cost" introduced in Sleator and Temperley [33] does not have a clear mathematical justification, but is used to denote grammatical graph structures with lower probabilities that induce the parser to construct parse trees with minimized cost. On the other hand, adaptive cost-based models [34] are used for query optimization in relational databases; they calculate the cost of various query execution plans based on the database table structures and the cardinality of the distribution of values among the table columns. In the case of probabilistic or non-axiomatic logic [13, 38, 39], a cost estimate can also be obtained based on the number and computational costs of multiplication and division operations applied to the corresponding number of terms involved in the inference.

The works by Wang[42], Wang et al. 43], and Isaev [17] propose an architecture for parallel execution of multiple inference tasks based on "budgeting," where each term within a task takes approximately constant computational time, and tasks, called "bags," are allocated computational budgets based on the task’s importance and urgency. Budgets are determined by the task’s priority in a multitasking environment and durability, which specifies the time at which it is expected to complete, so that the task is terminated at the designated time and reports the inference state reached at that time.

To provide greater control over such "budgeting," we define $\widetilde{C}_{ij}$ as the individual inference cost for agent *i* when evaluating $\widetilde{E}_{ij}$ for concept or action *j*. This defines a "personal cost" that could potentially prevent an agent from devoting its resources to analyzing a specific situation in favor of the least-cost options. According to the design of $\widetilde{E}_{ij}$, the cost of its evaluation can be estimated in advance and factored into the "budgeting" process, so that time- and quality-critical tasks with higher costs, known in advance, can be prioritized to produce high-quality results by the scheduled time. For a single terminal term $\widetilde{E}_{ij}$, the maximum cost of its computation can be estimated, according to the formula, as the cost of one multiplication $C_m$ of two weighted sums, plus the cost of two divisions $C_d$, plus the cost of $L + K$ multiplications $C_m$ within each sum, plus twice the cost of $L + K$ additions $C_a$ in the numerators and denominators of these weighted sums. For non-terminal terms $E^B_{ijl}$ and $E^S_{ijk}$, which would require recursive computation, assuming non-recurrent inference so that each term is evaluated only once, there will be three additional additions of the corresponding costs $C^B_{ijl}$ and $C^S_{ijk}$, computed recursively using the same formula given below.

$$\begin{aligned} \widetilde{C}_{ij} = \,C_m + 2*C_d + (K+L)*C_m + 2*(K+L)*C_a +  \sum _{l=1,L}(C^B_{ijl}) + \sum _{k=1,K}(C^S_{ijk}) \end{aligned}$$

That is, the computational cost of inference based on social evidence and believed evidence depends linearly on the size of the social graph and the number of belief items involved in the computation, and nonlinearly on the levels of recursion required to evaluate non-terminal terms such as "I believe he likes this kind of music."

Global cost estimates $\widetilde{C}_{ij}$ with respect to $\widetilde{E}_{ij}$, as well as specific cost estimates $C^B_{ijl}$ and $C^S_{ijk}$ with respect to $E^B_{ijl}$ and $E^S_{ijk}$, respectively, can be maintained based on various policies, including static cost caching with periodic system-wide cost updates or dynamic cost evaluation before each inference step to prioritize alternative inference flows. Specific cost estimates $C^B_{ijl}$ and $C^S_{ijk}$ can be made as a predefined constants for the terminal relations $E^B_{ijl}$ and $E^S_{ijk}$ present in the graph, or, in case such an estimate is not found in the graph and must be inferred, computed recursively along the tree of higher-level relations from top to bottom to the terminal relations at the bottom. Numerous variations of the cost estimation scheme may be considered, such as experimental determination of the cost of evaluating terminal relationships based on statistical observations of the costs of evaluating concepts, actions, and relationships, including the approval and disapproval relationships $E^B_{ijl}$ and $E^S_{ijk}$.

The formulas above are given for each individual agent *i*, so that each agent with such a graph can make inferences from the point of view of the entire community, either considering itself individually or from the point of view of any other agent in society represented in the "social graph", which allows one to take into account the full scope of the phenomena described by Cialdini [6]. This also allows one to run community-scale simulations, both for community management purposes and for individual purposes on behalf of an agent, as presented in Lefebvre [30], but using much more information about each agent’s beliefs. However, reducing the size of the graph by eliminating all agent-specific social graphs except the one surrounding the one specific agent with identity of "I" eliminates the need to index by *i*, reducing the graph size and the social computational cost by reducing accuracy of predictions and evaluations based on social evaluations.

Optimizing cost efficiency by reducing the memory footprint of elements in LTM and STM can be based on eliminating or pruning concepts or relations in the knowledge hypergraph in terms of their probabilities or costs, or both. According to Parr et al. [31], this pruning process would focus on eliminating less probable concepts in the belief system and world knowledge graph as well as actions at the forks of the procedural knowledge graph. However, when probabilities are unknown or equal, they can be decided to stay based on maximum social evidence for concepts known to social partners or actions that are expected to be liked by them, so that social evidence or socially derived probability can be used. Moreover, in the case where even social evidence is absent or equal, cost may be used, so the most expensive hypothetical concepts and possible actions can be eliminated from the focus of STM or forgotten entirely and removed from LTM.

A potentially promising hypergraph optimization criterion may be based on local maximization of probabilities with local minimization of costs within the cognitive model represented by the hypergraph, minimizing the global "freedom of transition" score defined in Wrenn et al. [44] and recognized as practical and related to minimization of Shannon entropy and maximization of information compression from the point of view of the development of language structure in Kolonin [26].

From a practical perspective, if a hypergraph contains a divergent action tree, then the number of possible subsequent actions in a given state corresponds to the freedom of transition from one state to another for possible states based on these actions. The decision-making process involves evaluating each option, so eliminating some of the options, which reduces the freedom of transition, also reduces the cost of choosing the correct solution by evaluating all possible options.

Optimizing cost efficiency by reducing overall *K* and *L* as discussed above and eliminating the levels of recursion needed to evaluate the reliability or preference of each piece of knowledge leads to the following. Each concept, action, or relationship has a better chance of being remembered if its probability is maximized and cost is minimized and it has a minimum number of links to other items of lower probability or higher cost. In terms of the hypergraph structure proposed above, uncertain knowledge from the "Imagination Graph" tends to be either "forgotten" from the "Imagination Graph" or "hardwired" into the "Foundation Graph" due to resource constraints. What is not "forgotten" or "hardwired" and remains "uncertain" in "Imagination Graph" is resolved using "social evidence" from the "Social Graph" whose items are recursively optimized based on the same resource constraints by "forgetting" weak social links and "hardwiring" strong ones.

The architecture and the model as a whole assume that the formation of knowledge accumulated in LTM during the life cycle of the system is loaded into it from STM, where all experiential evidence and planned actions are evaluated based on the formulas stated above and either rejected due to inconsistency with the core belief values and social context, or remembered and executed. Remembering concepts and actions in STM can be used temporarily to deal with more upcoming evidence and projected actions in the current operational context. Remembering in LTM allows for the reuse of concepts and actions when the the same or similar operational context is experienced. That is, the process of acquiring and executing knowledge is carried out as constant experiential learning and self-development during interaction with the physical and social environment under the pressure of resource constraints.

### Practical implementation

The main goal of this study is to substantiate an approach for accounting for non-factual evidence, such as "social evidence" and "believed evidence," in addition to the accounting for factual evidence considered in existing non-axiomatic and probabilistic logics [13, 38, 39], and to consider time and energy constraints not fully covered in the aforementioned studies. The proposed model and architecture can be implemented either as an extension of these frameworks or as an entirely new system. In the former case, it would add additional implementations of the "revision rule" to account for non-factual social and believed evidence. In the latter case, the new system, based on the "revision rule" presented above for social and inferential evidence, would be extended to support factual knowledge, as well as inference rules for "deduction," "induction," and "abduction," according to one of the options mentioned.

In any case, the resource consumption minimization principles described above can be applied either as add-on features in existing frameworks or as built-in principles in a completely new framework. Specific implementation aspects applicable in both cases are discussed below.

The computational model described above relates to a recursive reasoning mechanism on higher-order structures, linking belief elements and entities in a social graph in a recurring fashion ("I believe he likes it when she believes it"). The existence of such a recurrent knowledge structure, especially under the resource constraints discussed above, requires special controls to prevent cyclic computations, either preventing them entirely or ensuring that they do not run indefinitely. Two key options here are timed termination and caching.

The timed termination is suggested by Wang [42], Wang et al. 43] and Isaev [17], so that each task is completed after the budget allocated at its beginning has expired, and so that the best solution inferred so far is collected.

However, to prevent a situation where some nodes in the evaluation graph are not evaluated at all, evaluation results such as "strength" according to Goertzel et al. [13], "frequency" according to Wang [40], or "probability" according to Vityaev and Pak [37] can be cached so that once the computational task budget is exhausted, older values are collected instead of their evaluation proceeding to the next level of recursion.

In addition to the above, the evaluation cache can be timestamped so that completely outdated evaluations are ignored and recent evaluations are reused, preventing excessive redundant recursion based on some threshold of acceptable expiration.

The stress on available computing resources and allocated computing time can be proactively managed by estimating the cost and execution time of a task before scheduling it, using the model described above. In this case, if a low-priority task is expected to fail to complete on time, it can be avoided altogether to free up resources for more important tasks. Alternatively, the cost reduction process can be carried out in advance by setting limits on the amount of social and believed evidence based on thresholds that limit the number of elements in the social graph and belief (foundation graph) involved in the inference, as discussed in the previous section and illustrated by computational experiments in the next section.

## Empirical evaluation

The cognitive-behavioral model presented above can provide explanations for a wide range of specific behavioral phenomena and social dynamics described in Cialdini [6] and demonstrated in Dalege et al. [8]. It can also potentially be used in applications, briefly described in the next section.

**Fig. 7:**
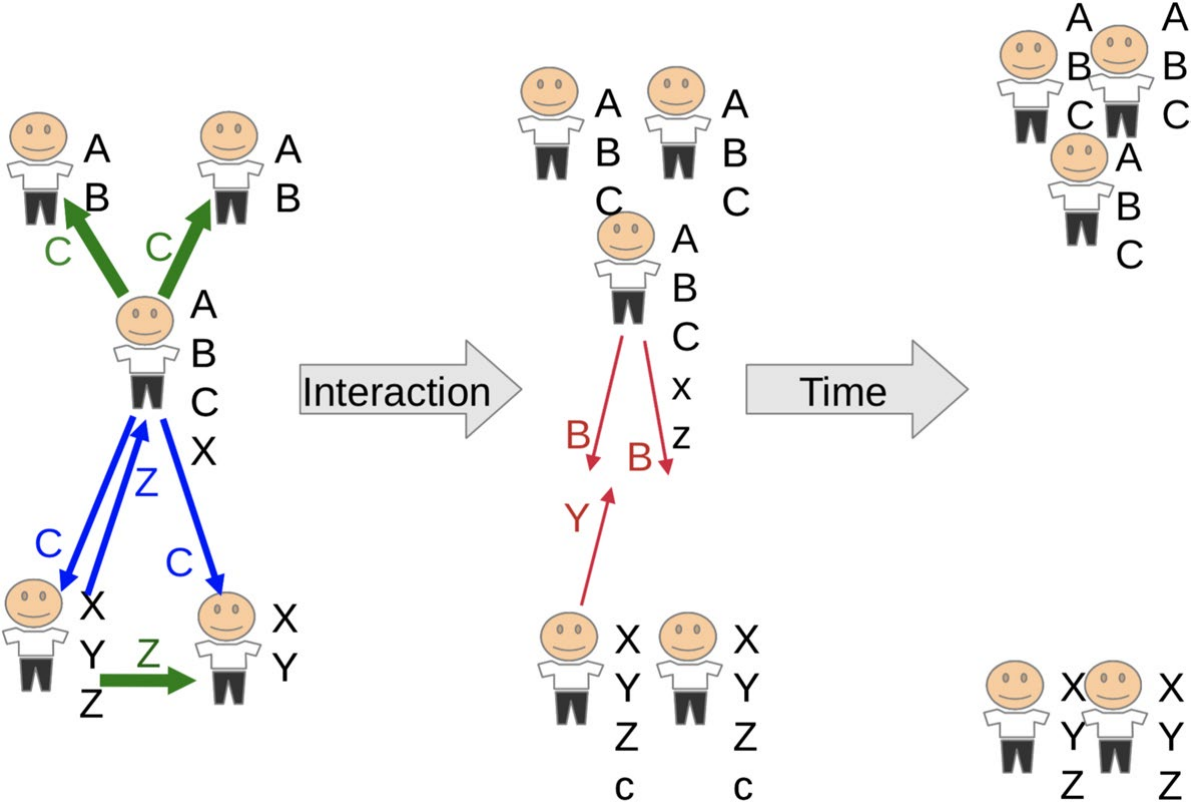
An example of the expected social dynamics driven by the presented model. Left: an initial configuration of five agents, some of whom communicate their belief items to other peers. Middle: after the communication, shared beliefs are adopted by participants based on their social proximity, so agents in mental proximity to one another become closer, leading to increased social polarization so that belief items communicated between the social groups are even not accepted further. Right: over time, due to resource constraints, unimportant belief items are forgotten, leading close agents to become even closer, causing further polarization

Below, we present two evaluations of the presented model: a qualitative imaginary experiment discussing the expected dynamics and a computer simulation confirming the expected dynamics quantitatively. The experiment is based on an imaginary community of five agents whose beliefs are represented by six imaginary recreational activities indexed with capital letters: A - Aikido, B - Basketball, C - Cricket, X - Xara, Y - Yoga, Z - Zumba, as shown in Fig. 7. The first two agents share their beliefs regarding A and B, the last two agents share beliefs regarding X and Y, with one of them possessing a unique belief element, Z. There is also a fifth agent in the middle who shares A and B with the first two and shares X with the last two.

### Expected dynamics

The example in Fig. 7 shows three sequential stages of multi-agent interaction, illustrating the expected impact of the model. Below we describe three stages of interaction and social structure in a community where different agents have different belief system items (A, B, C, X, Y, Z) and communicate with each other over time.

Stage 1 (Fig. 7, left): the community is connected by an agent in the middle who shares A and B with the agents at the top and shares X with the agents at the bottom, the agent in the middle broadcasts C to everyone, one agent at the bottom broadcasts Z to the agent in the middle and another agent at the bottom; C is well received (green) by the agents at the top due to high social ties between them and the agent in the middle based on shared A and B; C is poorly received (blue) by the agents at the bottom and Z is poorly received (blue) by the agent in the middle due to low social ties between them based on only X; Z is well received (green) by another agent at the bottom due to high social ties between both agents at the bottom based on shared X and Y.

Stage 2 (Fig. 7, center): agents in the middle do not perceive Z very well and subjectively evaluate X’s importance as low, so it is reduced due to low social support from agents in their social proximity; agents at the bottom do not perceive C very well, respectively; three agents at the top become socially closer and engage in a tighter communication cycle, same for agents at the bottom; the agent in the middle communicates B to the agents at the bottom, but it is not accepted at all (red), since it has a low social evaluation due to low social connection due to almost complete lack of belief overlap between the parties; similarly, Y, communicated by one agent at the bottom to the agent in the middle, is not accepted at all (red), due to low social connection between the agents.

Stage 3 (Fig. 7, right): over time, the agent in the middle loses residuals of X and Z in its belief due to lack of social support from other agents nearby; correspondingly, the agents at the bottom lose residuals of C due to relatively low relevance; eventually we find two isolated social clusters with identical beliefs within them and no overlap in shared social values between them.

This example represents the phenomena of how any initially heterogeneous society can diverge into disparate clusters unless there are external environment factors imposed on the society that create a common agenda that consolidates it. The model described above simply provides a low-level explanation of this dynamic in terms of the implementation of an inference mechanism based on what we call social evidence and resource constraints.

### Computational experiment

The multi-agent configuration described above was evaluated in a computational experiment using computer simulation, the code for which is available at https://github.com/aigents/pygents/blob/main/notebooks/social/social_dynamics.ipynb. In the experiment, five agents were assigned belief items regarding their imagined recreational activities, with the degree of belief, or mental attachment, set to 1.0 for preferred activities and 0.0 otherwise. The experiment itself involved three rounds of broadcast communication between agents, with different hyper-parameters corresponding to different strategies for reducing the computational cost of updating beliefs based on these messages.

In each round of the experiment, each agent shares its beliefs with the entire community, while other agents accept these beliefs as a weighted average according to the $\widetilde{E}_{ij}$ defined earlier, with the social reference $S_{ik}$ serving as a weight, calculated as the cosine similarity between the beliefs of two agents and interpreted as the social (mental) closeness between the beliefs of these agents at the peer-to-peer level.

The hyper-parameters explored were as follows.

- $recalc\_proximites=rare|frequent$—the evaluation of social proximities based on shared beliefs (calculated as the cosine similarity between beliefs) can be rare (social proximities are evaluated once per round) or frequent (all proximities are updated after each agent communicates).
- $peer\_threshold=0.0|0.5$—is the threshold value of a peer’s social proximity based on which it should be taken into account or ignored when updating beliefs based on incoming social data, while ignoring reduces computational costs by constraining the agent’s individual social graph *K*.
- $forgetting\_threshold = 0.0|0.5$—is the threshold value of a belief item that can be retained in the belief or forgotten if it is below the threshold, where forgetting reduces computational cost limiting the size of the agent’s personal belief (foundation) graph *L*.
- $communication\_order = natural|reverse$—is the order of broadcasting by agents during the experiment, used to assess the reliability of the experiment and to study the dependence on the broadcast order itself.

**Fig. 8:**
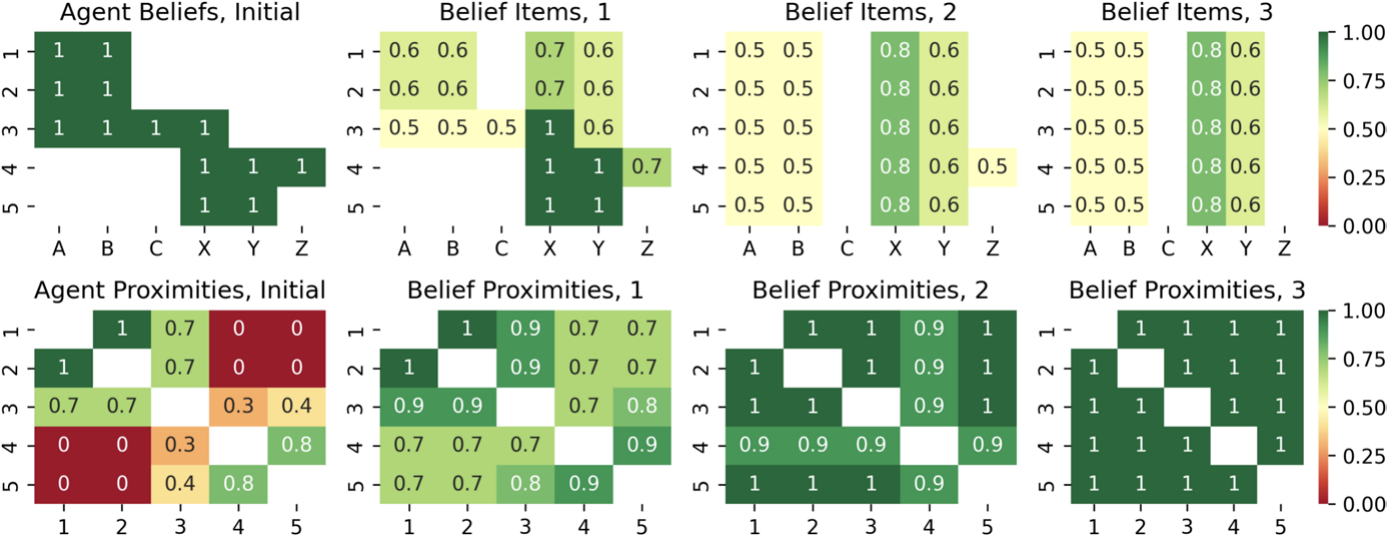
Results of three rounds of multi-agent simulation with the forgetting threshold $forgetting\_threshold = 0.5$, the social relationship threshold $peer\_threshold = 0.0$ (limiting belief capacity *L* without constraint on social connections *K*). Top row: values of belief items A, B, C, X, Y, Z for five agents 1, 2, 3, 4, 5. Bottom row: social proximity matrices between agents, estimated as the cosine similarity of their beliefs. From left to right: the initial state before the simulation, and then the updated states of beliefs and proximities after three subsequent rounds of multi-agent communication

**Fig. 9:**
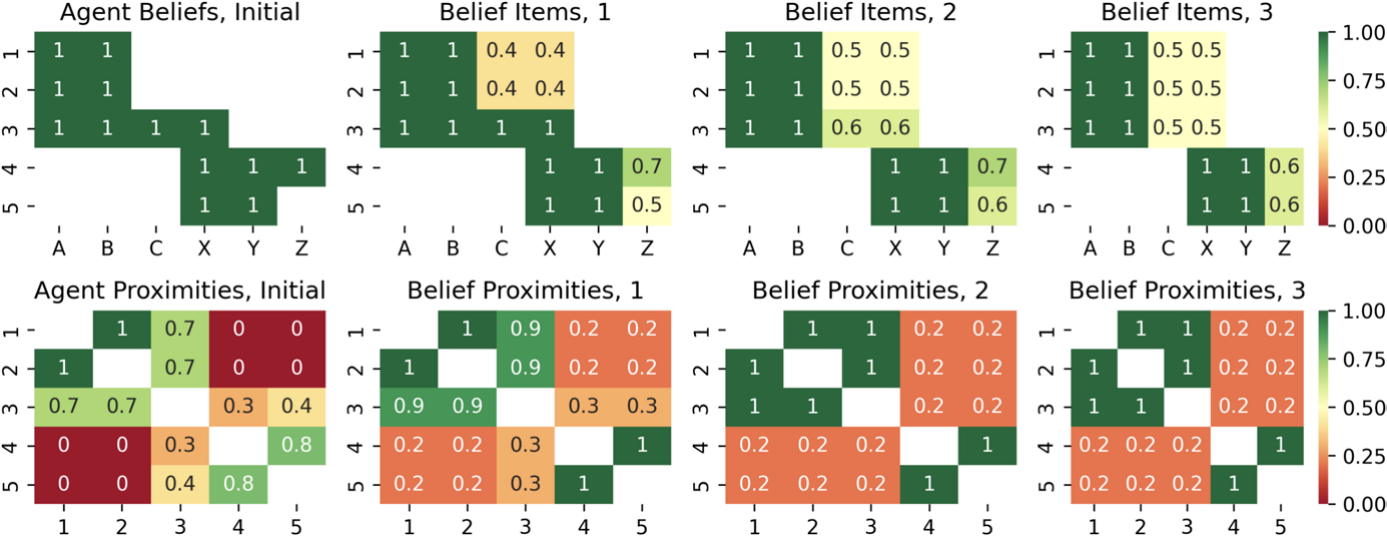
Results of three rounds of multi-agent simulation with the forgetting threshold $peer\_threshold = 0.5$, the social relationship threshold $forgetting\_threshold = 0.0$ (limiting social connections *K* without constraint on belief capacity *L*). Top row: values of belief items A, B, C, X, Y, Z for five agents 1, 2, 3, 4, 5. Bottom row: social proximity matrices between agents, estimated as the cosine similarity of their beliefs. From left to right: the initial state before the simulation, and then the updated states of beliefs and proximities after three subsequent rounds of multi-agent communication

Quantitative simulation experiments conducted using different hyper-parameters confirmed the model and qualitative analysis of the expected dynamics described above, with specific observations presented below and partially illustrated in figures Figs. 8 and 9.

- When agents are socially open and do not experience resource scarcity ($peer\_threshold = 0.0$, $forgetfulness\_threshold = 0.0$), they quickly agree on shared values and tend to form homogeneous communities with identical beliefs.
- When agents are not constrained by computational resources, allowing frequent updates of social proximity ($recalc\_proximites=frequent$), this leads to faster convergence of their beliefs than in the case of a more conservative and resource-efficient approach in which social references change rarely ($recalc\_proximites=rare$).
- When agents are stressed by a constraint on the effective belief size *L* ($forgetting\_threshold = 0.5$), the above unification also involves eliminating certain elements of the beliefs of agents in the minority, as shown in Fig. 8, which can be interpreted as a community-wide confirmation of the [2] experiment ("Asch conformity").
- In the case where agents are forced to minimize the effective number of social references *K* by reducing their communication circle to only socially close peers ($peer\_threshold = 0.5$), they tend to cluster into two different communities, demonstrating the social polarization described in the qualitative analysis presented above and shown in Fig. 7, confirmed experimentally as shown in Fig. 9. This can also be seen as a confirmation of the Asch conformity effect [2] for the smaller group, where agent number 3 (the agent in the middle in Figs. 7, 8, and 9) joins the community of the first two agents, losing its mental ties with the last two agents.
- The order of agents’ communication did not affect the results of experiments ending in polarization ($peer\_threshold=0.5$), but it did affect the results of experiments ending in unification ($peer\_threshold=0.0$), especially in the case of cost minimization based on belief size ($forgetting\_threshold = 0.5$). This confirmed the "recency effect" [15], such that the consensus of beliefs after unification was closer to the beliefs of the group of agents who spoke last. For example, in Fig. 8, the first pair of agents spoke first, but the unified belief throughout all rounds of communication was closer to the beliefs of the last two agents who spoke last. Changing the communication order during a round of the experiment changes the community’s belief structure in favor of the first two agents who spoke last.

## Applications of architecture and model

Based on the cognitive architecture and behavioral model presented above, we can expect the development of the following applications using it.

### Building socialized AI agents

In line with our original motivation, drawn from [5, 20, 22], and [11], the architecture and model can allow us to build AI agents with deep alignment to humans, interacting with them based on values embedded in robust and structured social graphs, rather than relying on potentially incomplete and contradictory sets of rules or vague masses of unstructured training data, as shown in Fig. 1. Our current work in this area is being done within the open-source project "Aigents" [23], dedicated to building a personal AI agent capable of capturing user interests, actions, social environment, and social interactions according to the architecture and model presented above and available as open source [24].

The current implementation of the "Aigents" agent allows extracting elements of the user’s belief system, social partners, and interactions in social and online media based on the endorsements given by the user to the agent using official APIs provided by social networks, messengers, forums, and blockchains such as Reddit, Twitter, Telegram, Slack, Discourse, Ethereum, Steemit, and Golos. The knowledge graph learned by the agent is used to recommend online content according to formulas and a corresponding inference engine that is part of an open source project referenced above.

### Social treatment and manipulation

**Fig. 10:**
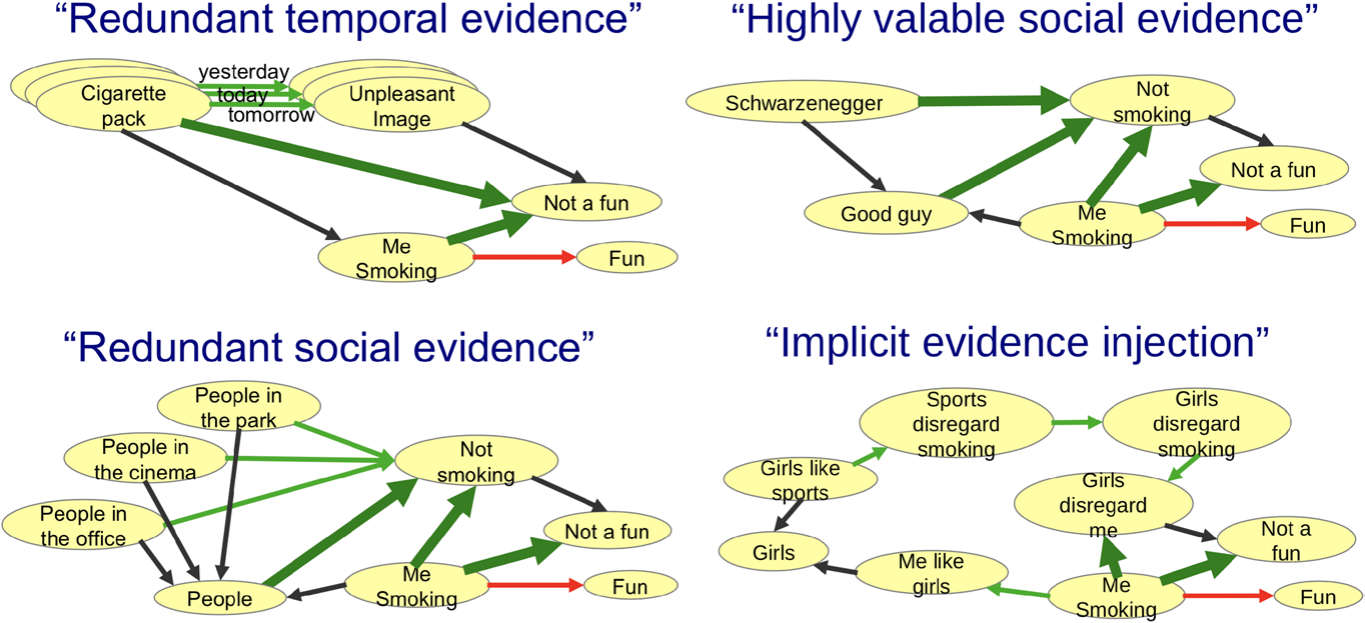
Examples of four basic belief manipulation techniques demonstrating the suppression of a person’s "smoking is fun" habit and its replacement with "smoking is not fun" habit. Top left: Repeatedly presenting the same evidence for the target concept to the person, making it easier for them to accept it as part of the belief rather than having to reason about it each time. Top right: Presenting target evidence associated with a social referee that is highly valued by the person, so that it is accepted on the basis of authority. Bottom left: Presenting evidence for the target concept associated with as many social connections in the person’s environment as possible, so that it is accepted through "social proof" (the "Asch conformity" effect). Bottom right: Pieces of evidence not directly related to the target concept is accepted one by one until a strong chain of evidence associated with the target concept is formed

The apparent side effect of our study has turned to be that the model which we present can explain social manipulation and engineering phenomena described by Cialdini [6] earlier. That can be used for wide range of belief manipulation applications for making good or preventing bad—for psychological treatment, which has to be supported or for social engineering which has to be resisted. Analysis of structure of the model and possible features of the inference process and processes of optimization of the knowledge graph structure targeted to meet the resources constraints can explain four basic belief manipulation techniques shown in Fig. 10, earlier described in Kolonin [21] and listed below.

*Redundant temporal evidence*—means providing redundant evidence of the same information to the target agent (a typical technique used by most high-end advertising channels).

*Redundant social evidence*—means delivery of the same evidence to the target agent through different communication channels through existing communities or communities created intentionally for this purpose (that is, the fundamental technologies of creating consumer communities and political parties).

*Highly-valuable social evidence*—means providing evidence through a highly trusted or reputable communication channel for the target agent (how "network marketing" works and why celebrities are actively involved in "brand marketing").

*Implicit evidence injection*—unlike the previous three techniques, which rely on brute force "massive evidence" or "social evidence", this is a way to change the portion of a belief that is reluctant about the incoming evidence if the information communicated is inconsistent with the current belief. In this case, the agent is presented with evidence, including social evidence, not explicitly related to the target concept, in sufficient quantity to get "hardwired" it in the agent’s "Foundation Graph." At some point, the agent is presented with some new evidence, including evidence with social proof, that directly links newly acquired knowledge to the target concept, promoting the expected behavior and suppressing the one targeted for elimination. At this point, the agent will change its target concept under the pressure of the entire chain of evidence and social evidence gradually loaded into its knowledge graph over time.

Figure 10 presents "good" example of belief manipulation by a psychologist helping a person to quit smoking and stop considering smoking a "Fun". Top left: "Redundant temporal evidence" by means of overloading "Evidence Graph" of an agent with repeatedly exposed evidence supporting the target concept of "Smoking not being a fan". Top right: "Highly valuable social evidence" by means of loading "Social Graph" of a person with a social reference with high value, also supporting the target concept. Bottom left: "Redundant social evidence" by means of overloading "Social Graph" of an agent with references supporting the target concept of "Smoking not being a fan". Bottom right: "Implicit evidence injection" by means of of loading "Social Graph" of person and its "Evidence Graph" with lots of aside information not directly relevant to the target concept, till the aside information propagates to "Foundation Graph" and then eventually present the connection of the injected socially supported and believed knowledge as connected to the target concept.

### Social modeling

Based on the observation that the presented model can be used to explain individual behavioral phenomena such as those discussed above, we suggest that large-scale modeling of social dynamics, such as that presented in Dalege et al. [8] and briefly illustrated at the end of the previous section, can be used for social and economic forecasting for business and social management purposes at various scales. For example, studying social dynamics to combat pandemics, as studied in Goertzel et al. [14], but relying on individual patterns of personal beliefs in the course of environmental and social communication, while explaining, predicting, and anticipating behavioral patterns across cultures under similar conditions.

## Discussion

We propose a cognitive architecture and behavioral model based on the principle of social proof or social evidence [6] and constrained by resources [40, 42, 43]. The architecture based on earlier high-level concepts [21, 29] consists of a knowledge graph segmented into functionally distinct subgraphs that are subject to specific probabilistic inference policies and supported by different storage types, an inference system, and two memory layers with dedicated content optimization functions. The inference system involves contextualizing knowledge, including perceived evidence and planned activity, based on core values in the belief system, referring to social value and reliability, if any. The assessment of social value is performed with reference to the social environment of the agent executing the inference mechanism, which may be a real social environment or imaginary social relations inferred by the agent. The inference process is determined by the costs associated with the specific knowledge to be inferred, given the time frame allotted for inference. The inference system can be based on one of the existing [13, 36–38, 43] or implemented from scratch.

The short-term and long-term kinds of memory provided by the architecture are supporting optimization of knowledge graph contents to fit available capacities on basis of maximization of usefulness of knowledge maximizing probability of its use, compactness and minimizing storage cost.

The behavioral model of an agent, whether an artificial system or a human simulation, involves mapping perceptual inputs from the environment to actions against the environment, based on evaluations of these actions by the inference engine in accordance with the social context and the underlying belief system, given the resource constraints imposed by the environment and the physical constraints of the agent. The basic belief system, in turn, is subject to constant change based on the same input data, social context, and resource constraints on the inference system and memory.

The architecture and model can be used to design and implement AI agents that match human values and experiences based on the alignment of their belief systems, capable of implementing decision support systems for practical applications. The model can also be proposed for computer modeling of human behavior individually or in groups, for psychological treatment, online security, and community management.

In our current and future work, we develop an AI agent based on the presented architecture, develop practical applications to assist in psychological treatment, and conduct large-scale social simulations for business and marketing scenarios. In particular, the following research directions appear valuable and promising.

- Although the earlier works [13, 17, 36–38, 40, 42, 43] discussed above offer promising alternative inference frameworks that deal with fuzzy "probabilistic" [13, 38] or "non-axiomatic" [40] knowledge, and some of them also include support for resource-based inference control, the "probability", "strength", or "frequency" measures in them are poorly aligned with each other and are defined differently in different terms. The assessment of the degree of uncertainty of these measures in the works themselves, called "confidence" [13, 17, 40, 42, 43], also differs. In our work, we attempt to justify the uncertainty measure based on what we call "social evidence" and "believed evidence" applied to the "revision" inference rule [42]. However, more fundamental and experimental research is needed to fully integrate all known inference rules, such as "deduction", "abduction" and "induction" [42] for factual knowledge with what we propose for non-factual knowledge.
- Additionally, while Isaev [17], Wang [40, 42], Wang et al. [43] offer a working solution for resource-based inference management, it can be improved for more efficient inference scheduling by prioritizing tasks based on inference cost estimates, taking into account the value of inferred elements in the context of the agent’s beliefs, as we propose. Research and development in such integration and practical evaluation of this approach is another promising direction.
- The previous attempt to build a cognitive architecture for experiential learning based on the "states" of an agent’s "core belief values" was made by Kolonin [25], and subsequent work [28] added the idea that energy efficiency is one of these "core values." Future work in this direction could be conducted by incorporating our concepts of inference cost estimation and possible approaches to its optimization presented in this study, as well as by evaluating the obtained solution on existing benchmarks for reinforcement learning [16] and experiential learning [46].
- The practical work on creating an artificial personal assistant based on the user’s beliefs and social environment, started in Kolonin [22–24], can be continued based on the computational and architectural design presented in this study.
- The practical application of psychological care methods, explored by Arinicheva and Kolonin [1], Bobo and Kolonin [4] in respect to pure diagnostic, can be expanded through behavioral modeling that anticipates the dynamics of change in a person’s beliefs under certain initial conditions and known social factors, which may allow for a more accurate diagnosis and, possibly, the provision of clinical recommendations. However, this direction clearly requires closer connection with field data and clinical validation.
- The social modeling capabilities provided by our model can be used to develop practical applications in marketing, corporate management, and community governance, as was done to analyze consumer and fraud behavior in Kolonin et al. [27], but with greater accuracy by taking into account dynamic beliefs, the social environment, and resource constraints.

## Conclusion

The cognitive architecture and behavioral model presented in our work can be considered novel in the following aspects.

First, we formalize the account of believed and social evidence ("social proof") for non-factual knowledge, complementing the factual knowledge considered in existing non-axiomatic and probabilistic inference frameworks.

Second, we formalize the account of resource constraints by estimating the cost of inference on such factual and non-factual knowledge and propose principles for its optimization.

Third, we propose the memory architecture for designing knowledge-based inference systems that can potentially implement the proposed principles.

Fourth, in the context of social psychology and multi-agent applications, we present an empirical evaluation of our architecture and model, confirming behavioral patterns previously known in the literature on human psychology and social science.

Finally, we propose possible applications that can be developed based on our model and architecture, and outline directions for future work.

## Data Availability

No datasets were generated or analysed during the current study.

## Abbreviations

AI: Artificial Intelligence
LLM: Large language models
LTM: Long-term memory
RAM: Random-access memory
STM: Short-term memory
TF: Transition freedom or freedom of transition
